# Medical school origins of award-winning pathologists; analysis of a complete national dataset

**DOI:** 10.1186/s12909-024-05790-8

**Published:** 2024-07-29

**Authors:** Sinclair Steele, Gabriel Andrade, Marwah Abdulkader, Yehia Mohamed

**Affiliations:** 1 College of Medicine, Ajman University, University Street, Al Jerf 1, Ajman, UAE; 2 King Fahad Specialist Hospital, Department of Pathology, Dammam, Saudi Arabia; 3 Department of Microbiology and Immunology, Faculty of Pharmacy (boys), Al-Azhar University, Cairo, Egypt

**Keywords:** Pathologists, Medical schools, Medical careers, Award-winners, Globalization, International Medical graduates

## Abstract

**Background:**

The ultimate aim of medical education is to produce successful practitioners, which is a goal that educators, students and stakeholders support. These groups consider success to comprise optimum patient care with consequently positive career progression. Accordingly, identification of the common educational features of such high-achieving doctors will facilitate the generation of clinical excellence amongst future medical trainees. In our study we source data from British clinical merit award schemes and subsequently identify the medical school origins of pathologists who have achieved at least national distinction.

**Methods:**

Britain operates Distinction Award/Clinical Excellence Award schemes which honour National Health Service doctors in Scotland, Wales and England who are identified as high achievers. This quantitative observational study used these awards as an outcome measure in an analysis of the 2019-20 dataset of all 901 national award-winning doctors. Where appropriate, Pearson’s Chi-Square test was applied.

**Results:**

The top five medical schools (London university medical schools, Aberdeen, Edinburgh, Oxford and Cambridge) were responsible for 60.4% of the pathologist award-winners, despite the dataset representing 85 medical schools. 96.4% of the pathologist merit award-winners were from European medical schools. 9.0% of the pathologist award-winners were international medical graduates in comparison with 11.4% of all 901 award-winners being international medical graduates.

**Conclusions:**

The majority of pathologists who were national merit award-winners originated from only five, apparently overrepresented, UK university medical schools. In contrast, there was a greater diversity in medical school origin among the lower grade national award-winners; the largest number of international medical graduates were in these tier 3 awards (13.9%). As well as ranking educationally successful university medical schools, this study assists UK and international students, by providing a roadmap for rational decision making when selecting pathologist and non-pathologist medical education pathways that are more likely to fulfil their career ambitions.

## Background

To individuals outside the profession, the word ‘pathologist’ conjures up images of doctors performing autopsies on deceased patients [1] in an attempt to discern the cause of death. In reality, this is only a small part (approximately 5%) of the routine work of most pathologists. The vast majority of a pathologist’s time is spent in diagnostic endeavours. For example, examining tissue samples to diagnose or manage cancer, reviewing cervical Pap (Papanicolaou) smears, rapid examination of surgical specimens and presenting pathological information at multidisciplinary team meetings are more reflective of pathologists’ activities. This technical discipline is scientifically demanding and requires a foundation in training that commences in undergraduate medical degrees. Furthermore, it has historically been accepted as a truism in *medical training as the science of disease* that underpins the majority of subsequent clinical disciplines. In fact, in the UK the Royal College of Pathologists refers to pathology as the “*science behind the cure*.” Students or trainee doctors that are interested in being well-trained doctors or wish to become pathologists often seek out medical schools/colleges that have robust embedded pathology training. One measure of the effectiveness of the pathology training is the production of successful clinical pathologists. Accordingly, our project examines the educational backgrounds of successful clinical pathologists.

Historically, in Britain there have been two clinical merit award schemes established to reward successful clinicians employed in the National Health Service (NHS):

(i) The Clinical Excellence Awards (CEA) scheme, covering Wales and England [2].
(ii) The Distinction Awards (DA) scheme, covering Scotland [2].

The schemes are similar in aims and organization; both offer tiers of local and national awards to high-achieving doctors. However, the CEA scheme is currently being restructured, renamed and re-established as the National Clinical Impact Awards (NCIA), whilst the DA scheme remains in place in Scotland. The doctors receiving such awards gain benefits not only from the effects of these honours on their reputations and career progressions but also from the recurring financial rewards accompanying such accolades [2].

These UK national award schemes were envisioned and implemented after World War II for the pragmatic purpose of motivating senior clinicians to support the newly-created NHS. Since their inception, these schemes and their implementation have been the cause of vigorous debate in the UK medical community. As a result, these clinical merit awards have been evaluated and discussed from the perspective of award objectivity [3], specialty distribution [4], regional distribution [4], gender parity [2], age distribution [5] and ethnicity/racial distribution [6] but, until our research series, *not by medical school of origin*. These constructive criticisms have resulted in iterative revisions of these award schemes over the previous three decades. Many medical commentators agree that there should be a system to reward high-achieving clinicians [7] and the CEA/DA/NCIA merit awards are seen as national recognition of clinical career success - accounting for their continuing value, greater than 70 years after their inception. This original innovative research study is part of a series that contributes to the medical education discussion by relating the pathologist and non-pathologist merit award-winners to their *medical schools of origin*. We place our findings in the contexts of educational, career and global implications for ambitious prospective medical students, undergraduate medical students and doctors aspiring to attain career success [8, 9].

## Methods

The lists of pathologist award-winners and non-pathologist award-winners were retrieved from the source material of the DA annual report (Scotland) for 2019–2020 [10] together with the CEA annual report (England and Wales) [11] for the 2019–2020 awards round. These lists were summations of both the newly selected awardees and the previous award-winners who had retained their awards. The medical schools of origin were identified by using the published Medical Register, UK [12] as well as the published Dental Register, UK [13–15].The total number of award-winners was 901 - the university medical schools of origin were successfully identified for 99.8% of these clinicians [14, 15]. Accordingly, 899 doctors were included in the analyzed dataset. Award-winning doctors in the publications above, who were designated as specializing in the core pathological disciplines, were included in this study [14, 15]. In the 2019-20 award round the following specialties were included: (general) pathology/forensic pathology and histopathology [14, 15].

The rankings of medical schools by number of merit award-winning alumni were determined by summation of the number of pathologist award-winners of A plus (A^+^), A or B grade (or platinum, gold, silver or bronze award-winners) [14, 15]. Only these national level Clinical Excellence Awards and Distinction Awards were included in this study [14, 15]. Combining these parallel and similar award gradings, permitted all of Britain’s (England, Wales and Scotland) excellence award-winners to be analyzed together [14, 15]. As part of our analysis of the grades of awards we collated the award categories to explicitly show the three tiers of national merit awards; A plus and platinum award-winners were combined to yield the top tier (tier 1) of national pathologist awards [14, 15]. The A and gold awards were combined to create the intermediate tier (tier 2) of national pathologist awards [14, 15]. Finally, the B and silver/bronze awards were combined to create the lowest tier (tier 3) of national pathologist merit awards [14, 15]. The same approach was taken with the non-pathologist data [14, 15].

The rankings of the medical schools by the number of merit award-winning alumni were approximately size corrected by dividing the total number of award-winners that were alumni of the medical school by the number of admissions to the undergraduate medical school in the 2019-20 academic year [14, 15]. We used this pragmatic approach to estimate the size correction rather than the more ideal but inaccessible integral of medical school graduation numbers against time for approximately the last 50 years [14, 15]. The comparison of the distributions of award-winners (pathologist merit award-winners versus non-pathologist merit award-winners) was quantified using Pearson’s Chi-Square test with the significance level set to *p* < 0.05 [14, 15].

On the basis of the frequency of award holders in the 2019-20 round, the top 20 medical schools were selected. For those 20 medical schools, a Pearson’s coefficient was calculated to determine the correlation between the age of the medical school by establishment date and the number of award-winners corrected by size (award- winners/number of admissions 2019-20).

All procedures were performed in compliance with the pertinent guidelines [14, 15].

Patients and public involvement; no patient involvement [14, 15]. The methods that were applied in our study, and that cover the description in this methods section, were similar to and closely derived from earlier publications in this series, which we cite here [14, 15].

## Results

The 55 core pathologists indicated in the 2019-20 award round represent not only the new award holders but also the cumulative total of all pathologist award-winners in that year together with all previous years, at the time of publication. The largest category was designated (general) pathologists amounting to 70.9% of all the merit award-winning pathologists.

Table 1 shows the ten medical schools that attained the largest number of alumni merit award-winners; these award-winners possessed platinum, gold, silver, bronze, A plus, A or B awards. More importantly, Table 1 compares the originating medical schools of the pathologist and non-pathologist merit award-winners for the ten medical schools with the largest numbers of award-winners; the table contrasts the numbers and percentages of pathologist award-winners and non-pathologist award-winners which the graduates of each medical school attained. Pearson’s Chi-Square test demonstrated a statistically significant difference between the distributions of the medical schools of origin for pathologist merit award-winners versus the non-pathologist merit award-winners, *p* < 0.01 (*p* = 0.005, Chi-Square 12.91). Graduates of London university medical schools, Aberdeen, Edinburgh, Oxford and Cambridge medical schools accounted for 60.4% of pathologist award-winners. In comparison, 53.3% of the non-pathologist merit award-winners were graduates of five British medical schools: Aberdeen, Edinburgh, Glasgow, London university medical schools and Oxford.

Table 2 displays the effect of the approximate medical school size correction on the ranking of the medical schools by number of alumni award-winners. London’s number one ranking (pathologists) before size correction dropped to a number seven ranking after size correction. Similarly, London’s number one ranking (non-pathologists) before size correction became a number seven ranking after size correction.

Our analysis included a comparison of the pathologist A plus/platinum award-winners (designated tier 1) with A/gold award-winners (designated tier 2) and B/silver/bronze award-winners (designated tier 3). The tier 1 pathologist award-winners came from 6 medical schools: Belfast, Edinburgh, London, Oxford, Sheffield and Southampton. The tier 2 pathologist award-winners came from 8 medical schools: Aberdeen, Belfast, Birmingham, Dublin, Edinburgh, Glasgow, Ireland (Royal College of Surgeons) and Oxford. The tier 3 pathologist award-winners originated from 17 medical schools: Aberdeen, Birmingham, Bologna, Cambridge, Edinburgh, Glasgow, Goethe, Ireland, Leeds, London, Manchester, Mysore, Nottingham, Osmania, Oxford, Sheffield and Tirana.

Table 3 contrasts the continental locations of the originating medical schools for pathologist and non-pathologist merit award-winners; for the ten medical schools with the greatest numbers of award-winners. 96.4% of pathologist merit award-winners were from European medical schools, in comparison 91.4% of the non-pathologist award-winners were from European medical schools. Pearson’s Chi-Square test indicated that there was not a statistically significant difference between the continental locations of the originating medical schools for pathologists and non-pathologist merit award-winners, *p* > 0.05.

After evaluating the top 20 university medical schools (arranged on the basis of award-winners’ frequencies), a moderate and positive correlation was found between the age of the medical school by establishment date and the number of award-winners corrected by size (number of admissions), r [18] = 0.47, *p* = 0.04.

11.4% of all the merit award-winners were international medical graduates (IMGs) - meaning that they were not graduates of UK or Irish medical schools. 9% of the pathologist award-winners were IMGs. The pathologist tier 3 award-winners included the greatest proportion of IMG award-winners at 13.9%.

**Table 1:** Top 10 medical schools; analysis by number of pathologist award holders, number of non-pathologist award holders and total number of award holders

Medical school	Total number of award holders	Number of pathologist award holders	Percentage of pathologist award holders	Number of non-pathologist award holders	Percentage of non-pathologist award holders
London	179	10	18	169	20.02
Glasgow	113	3	5.5	110	13.03
Edinburgh	84	4	7.3	80	9.48
Aberdeen	60	6	11	54	6.40
Oxford	45	8	15	37	4.38
Cambridge	43	5	9.1	38	4.50
Manchester	38	1	1.8	37	4.38
Birmingham	29	2	3.6	27	3.20
Dundee	29	0	0	29	3.44
Nottingham	26	1	1.8	25	2.96

**Table 2:** Top 10 medical school rankings by number of graduates holding merit awards; with or without estimated size correction

Medical school	Total number of pathologist award holders	Ranking by number of pathologist award holders	Ranking by pathologist award holders after size correction	Total number of non-pathologist award holders	Ranking by number of non-pathologist award holders	Ranking by non-pathologist award holders after size correction
Glasgow	3	6	5	108	2	1
Edinburgh	4	5	3	78	3	2
Oxford	8	2	1	37	6	3
Aberdeen	6	3	2	54	4	4
Dundee	0	10	10	29	8	5
Cambridge	5	4	4	38	5	6
London	10	1	7	167	1	7
Manchester	1	8	9	37	7	8
Birmingham	2	7	6	27	9	9
Nottingham	1	9	8	25	10	10

**Table 3:** A geographical comparison of the medical schools of origin of pathologist and non-pathologist merit award holders

Continental location of medical school	Non-Pathologists		Pathologists	
	Total number of non-pathologist award holders	Percentage of total number of non-pathologist award holders	Total number of pathologist award holders	Percentage of total number of pathologist award holders
Africa	19	2.25	0	0.00
Asia	39	4.62	2	3.64
Australasia	9	1.07	0	0.00
Europe	771	91.35	53	96.36
North America	5	0.59	0	0.00
South America	1	0.12	0	0.00
Total	844	100%	55	100%

## Discussion

### Pathologist merit awards and UK medical schools

Our study is part of the first series to comprehensively analyze British clinical merit award-winners’ medical schools of origin. This project identifies medical schools that have facilitated the successful medical education of pathologists by using the outcome measure of clinical merit award-winning. As a result, the data and analysis we provide will be of significance to local potential medical students as well as current and future graduates of International Medical Programs [16]. Our series of studies are the first to *rank medical schools by the number of merit award-winners* originating from each school, and accordingly will provide a new comparative perspective for medical educators.

The UK has long been known to attract international medical graduates to practise medicine. This was further confirmed and quantified in the General Medical Council 2019 workforce study that stated “For the first time, more non-UK medical graduates took up a licence to practise than UK medical graduates.“ [17] As a result of such workforce migrations, the scope of possible medical schools of origin of merit award-winners has essentially become global. Specifically, our database of merit award-winners covering the 2019-20 round has 85 different medical schools represented. This study shows that after being chosen by a “transparent and defensible” assessing and scoring arrangement [18] 60.4% of the pathologist award-winners received their undergraduate training at one of only five UK medical schools (Table 1). These were London university medical schools, Aberdeen, Edinburgh, Oxford and Cambridge. A similar pattern of concentration occurred amongst the non-pathologist merit award-winners; 53.3% of these were graduates of Aberdeen, Edinburgh, Glasgow, London university medical schools and Oxford. The observation that there is a similar concentration of award-winners amongst graduates of similar medical schools, for both the pathologists and non-pathologists, implies that there may be common underlying non-specialty specific factors which account for the success of these doctors. The quality of undergraduate medical education may well be such a factor.

A Pearson’s Chi-Square test showed a statistically significant difference between the distributions of the medical schools of origin for pathologist merit award holders versus the non-pathologist merit award holders (*p* = 0.005, Chi-Square 12.91). Specifically, the successful pathologists were 2.6 times more likely to be graduates of Oxford or Cambridge university medical schools than non-pathologists. Considering the data presented in Tables 1 and 2 (whether or not a size correction is applied) the top four medical schools of origin of the pathologists include Oxford and Cambridge, so in this instance the prestige and good quality of medical education would seem to coincide in these universities [19]. Interestingly and in contrast, the high rankings of Glasgow and Aberdeen medical schools amongst non-pathologist merit award holders implies that a prestigious medical school alone is not as dominant a factor in the successful career development of non-pathologists. Based on our data, a strong local or international student candidate applying to medical school who has a desire to specialize as pathologist could be advised to favour Oxford, Edinburgh and Cambridge medical schools, whereas a less strong student applicant who definitely did not want to specialize as pathologist might be wiser to prioritize Glasgow medical school. A student who was not sure whether a pathologist or non-pathologist career pathway was preferable might consider Aberdeen medical school. Thus, the rankings of medical schools that we produced in this study provide data which future prospective medical students can use to select medical schools appropriate for their ambitions. Students generally make rational decisions in the field of education [20, 21] and ranking information of this type is particularly important to an educational pathway as complex and tortuous as attempting to train to be a doctor in a particular specialty. Recent studies have demonstrated that the differences between medical schools tend to remain stable over time [22], so the guidance offered here will have valuable longevity.

Our observations regarding the concentration of award-winning pathologists and non-pathologists within a comparatively small number of UK medical schools led to an examination of the role of medical school size on our award rankings. Specifically, after aggregation of the number of annual graduates, London medical schools effectively become one of the largest medical schools in Europe. Thus, as a percentage, London university medical schools’ alumni are likely to be well represented in any essentially Eurocentric medical award schemes. In order to investigate this consideration, we carried out an approximate size correction on the medical school rankings by number of award-holders, as indicated and discussed in the Methods above, using the 2019-20 university medical school admission numbers. After applying this approach to the pathologist award-winners rankings, the combined London university medical schools fell from the number one position prior to the estimated size correction to seventh position after size correction. A similar and parallel effect was noted when the size correction estimate was applied to the non-pathologist award-holder rankings; here combined London university medical schools fell from first to seventh in the rankings. Obviously, medical school size has an effect on the medical school ranking. However, it is unlikely that size alone accounts for the concentration award-holders in a small number of medical schools; factors related to the quality of the undergraduate medical training are entirely consistent with our findings.

### Pathologist merit awards and international medical schools

In view of the tendency of medical trainees and students to travel internationally in this era of globalization [23, 24] we also evaluated the originating medical schools of the award-winners by continent of location. Table 3 depicts the comparison of the originating medical schools for pathologist and non-pathologist merit award-winners. 96.4% of the pathologist award-winners originated from European medical schools whereas 91.4% of the non-pathologist award-winners were originally trained in European medical schools. Statistically, there was no significant difference between the continental locations of the originating medical schools for the pathologists and non-pathologists, in terms of their distributions, *p* > 0.05 (Chi-square test).

This study demonstrates a greater diversity of medical school origins among the lowest tier of merit award-winners than the highest tier of merit award-winners. Specifically, pathologists with tier 1 awards came from 6 medical schools representing one continent whereas tier 2 award-winners came from 9 medical schools representing one continent. In contrast, the tier 3 award-winners originated from 17 medical schools representing two continents. These findings would seem to indicate a trend towards greater globalization and inclusivity effects in the lower tier merit awards. The finding that the largest concentration of IMGs, 13.9%, was found among the lowest tier of award-holders also supports this observation. The larger number of lower tier awards and the shorter time required to achieve these lower grade awards than the higher tier awards, would understandably reveal such demographic trends more readily amongst the lower grade merit awards. Future longitudinal analyses of merit award-holders would be important in accurately determining whether this diversity trend progresses into the higher tier and more prestigious clinical merit awards.

### Merit awards; undergraduate and postgraduate training of pathologists and non-pathologists

This research project is unique in investigating the relationship between national award-winning pathologists and their originating medical schools. Specifically, little peer reviewed work has been published that investigates the effectiveness of each medical school in training their students and relates this to the future postgraduate success of each medical school’s alumni. We were only able to identify three authoritative studies [22, 25, 26]. The MedDifs study by McManus et al. [22] was the most comprehensive and included some components that were comparable to our study. The MedDifs study involved examining UK medical school performances using 50 different criteria that were either quantitative or qualitative in nature. These criteria were grouped into categories [22]:

- Selection of applicants.
- Student satisfaction.
- Curricular influences.
- Fitness to practise.
- Choice of training specialty.
- Postgraduate examination performance.
- Foundation entry scores.
- Perception of Foundation Year 1.
- Teaching/learning and assessment.
- Institutional history.

In comparing our study to the MedDifs study, we were obviously more limited in the number of factors pertinent to medical education that we considered and we followed a purely quantitative approach to the research. Unsurprisingly, McManus et al. were able to correlate their range of factors and reveal educational relationships. For example:

- Medical schools that focused on Problem Based Learning tended to produce doctors that scored lower in postgraduate exams.
- Doctors from the bigger medical schools tended to score worse in postgraduate exams.
- Medical schools that focused on self-regulated learning produced doctors that tended to perform better in postgraduate exams.

Both their study and ours shared the limitation of not being able to assess and compare medical school courses in undergraduate medical degrees. Furthermore, the MedDifs project was much more limited in its ability to identify causal relationships between its investigated educational factors.

In order to investigate the possible causalities in our presented medical school rankings for pathologist, non-pathologist and all merit award-winners (Table 1), we reviewed the histories of the UK medical schools [27–36]. We noted that all seven of the oldest medical schools in the UK, measured by establishment date, were present in our top 10 medical school rankings by award-winners for pathologists and non-pathologists. These were all established prior to 1826 and were Birmingham (1825), Manchester (1824), Aberdeen (1786), St Bartholomew’s university (1785), Glasgow (1751), St George’s London University (1733) and Edinburgh (1726) medical schools. Moreover, Oxford medical school was known to have been teaching medicine since the 12th century and Cambridge had been teaching medicine since 1524; in essence, these two medical schools had been teaching clinical disciplines before the formal establishment process had even been formed. Accordingly, it can be stated that of the top 10 medical school rankings (Table 1), *8 are the oldest medical schools in the UK.*

Furthermore, none of the more modern medical schools (established after 1999) are represented in our top 10 medical school rankings (Table 1). So, Warwick (2000), Norwich (2000), Peninsula (2000), Brighton and Sussex (2002), Hull York (2003), Keele (2003) and Swansea (2004) are not represented our top 10 (or top 20) medical school award-winner rankings. Whilst it may be understandable that the younger medical schools established within the last ten years may not yet have had time for their alumni to distinguish themselves to national merit award levels, it is less clear that this explanation accounts for the dearth of top 10 ranked medical schools established around the year 2000.

*In summary*,* our observations are consistent with at least a correlation between medical school age and the number of subsequent graduates becoming merit award-winners.* Furthermore, on evaluating the top 20 university medical schools a moderate and positive correlation was found between the age of the medical school by establishment date and the number of award-winners corrected by size, r [18] = 0.47, *p* = 0.04.

**Fig. 1:**
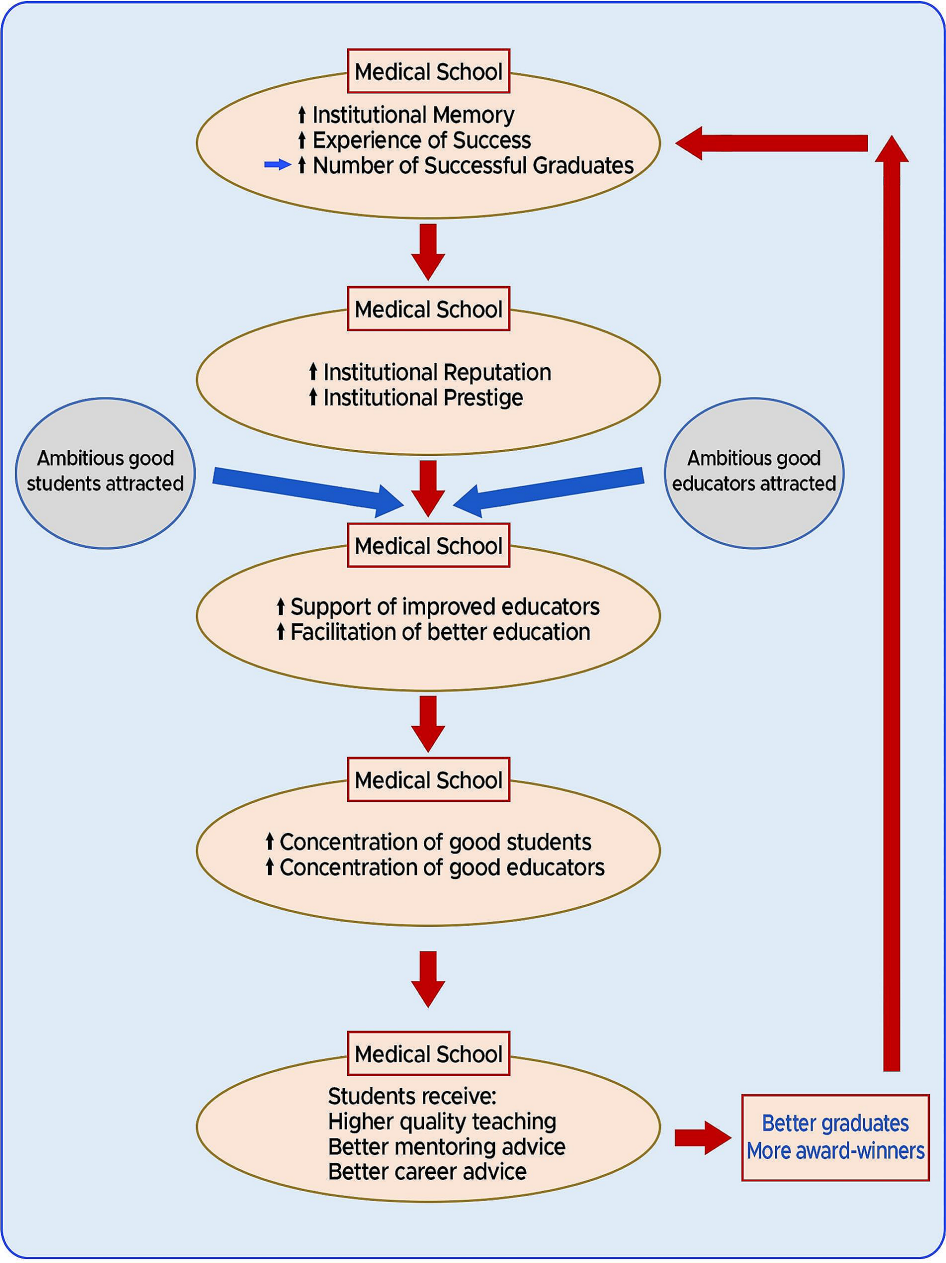
A model for the creation of award-winners. *Cycles of institutional memory and experience*

After considering the totality of the results of our research study and also accepting the previous results of the studies into UK medical school education [22, 25, 26], in Fig. 1 we reiterate a model first described, elucidated and published earlier last year [14, 15] - a model accounting for the age-dependent differential medical school performance in creating award-winning pathologists:

### Cycles of institutional memory and experience

1. Because of their greater age, the older university medical schools have accrued more **institutional memory and experience** in medical education than the more youthful medical schools. Accordingly, the older medical schools have a better chance of generating successful graduates - potentially before some of the younger medical schools have even become established.
2. As the older university medical schools appear to produce larger numbers of obviously successful alumni, they will garner positive reputations and inevitably be designated as more prestigious institutions. Consequently, **ambitious**,** competent** and career-focused students are more likely to be apply to these university medical schools.
3. Having produced more successful students, these older university medical schools will also have accumulated **more experience in positively managing and educating** such high-achieving students. Such experience will also coincide with improved **support** for the **better educators** in the medical school.
4. As a result, these older university medical schools with greater institutional memory and experience will tend to progressively and steadily **accumulate** a greater percentage of the **most able students and educators**.
5. Ultimately, the students in these older medical schools will tend to receive and benefit from **better quality teaching**, **better mentoring** and better medical **career advice**.
6. Thus, these older university medical schools will produce better educated, better advised and better prepared doctors who are more likely to become merit award-winners. There will also be an additional benefit to the originating medical school of having trained such high-achievers; they will accumulate greater **experience** in training award-winners, so adding to the **institutional memory** of successful education. *The cycle will then repeat.*

The medical education consequences of the action of *Cycles of Institutional Memory and Experience* can be described as follows:

- An inevitable result of the operation of the adjacent cycle is that the longer established medical schools have naturally experienced more cycling during their longer existences. This causes an accumulation of an increasing number of award-winners in the medical community, from each such originating medical school.
- The differential accumulation of award-winners in the community from each medical school depends on the relative efficacy and efficiency of the cycle in each medical school. Such efficiency differences account for the ultimate medical school rankings.
- The same considerations that led to development of the Cycles of Institutional Memory and Experience can also apply to the college/departmental/faculty levels. Specifically, a department that generates merit award-winning pathologists will tend to generate more such pathologists in the future. In principle, this could be termed a departmental cycle of memory and experience.

Any award scheme designed and administered by human beings runs the risk of introducing biases, thus leading to overrepresentation of particular groups. Our model provides a natural explanation and mechanism for connecting excellence/success with such bias. With every cycle of our model, increasing numbers of successful graduates originating from the older universities accumulate in the UK medical community. Subsequently, such distinguished and visible alumni are more likely to be elevated to senior leadership or managerial positions. These positions would include clinical excellence/distinction award allocators. Consequently, explicit selection biases or implicit selection biases would have a tendency to favour the graduates of these same medical schools of origin - resulting in a disproportionate number of these alumni gaining awards. Ultimately, we believe our model of *Cycles of Institutional Memory and Experience*, at least in part accounts for the concurrence of appropriate success/excellence in award-winning and apparent bias in our medical school rankings. Accordingly, it seems inevitable that the effects of genuine appropriate award attainment and bias are linked and would tend to be expressed simultaneously.

In the last year there has been a reorganization of the UK national clinical excellence scheme. Specifically, in January 2022, it was announced that the latest iteration would be termed the “National Clinical Impact Awards, NCIA.” [37] The governing authority announced that the objectives of this scheme would be to:

- Widen access.
- Simplify the application process, attempting to make it more equitable and inclusive.
- Reward excellence in a wider range of activities and behaviours. [38]

This new rewards scheme offers a natural test and challenge to our *Cycles of Institutional Memory and Experience* model. Our model is based on the history and epidemiology of medical education in the UK. Accordingly, an analysis of the medical schools of origin of the NCIA winners should yield rankings similar to those reported in our series of publications, assuming that there is an underlying value to the model. We look forward to testing our model in this way.

### Study limitations

Most of the traditional limitations of a study of this type have been implied and exemplified in our discussed comparison with the MedDifs study. We could not quantify the relative effects of postgraduate versus undergraduate professional circumstances on ultimate award-winning likelihoods. For example, a graduate of a less renowned medical school may move on to work in a successful and high profile research institute or specialized clinical settings - thus increasing their personal chance of attaining a merit award, beyond that which might have been predicted from their alma mater. We would emphasize that the medical school of origin is only one of many factors that determines ultimate career success and potential award-winning. It would be interesting to find out whether such postgraduate centres were themselves smaller scale centres of institutional learning and experience.

We also could not quantify the probable effect of the assessment criteria used for award giving, on the number of merit awards attained by specific medical schools. Undoubtedly, there is certain to be such an effect, however the dynamically changing nature of these assessment criteria since the inception of the awards in the post-World War II era make measuring such an effect more than challenging and beyond the scope of this study. In fact, the latest iteration of the merit awards, NCIA, has been designed to include a larger range of activities to measure excellence and to widen accessibility. Our study is better placed to show the different apparent attainments of the medical schools than the summation of all of the medical education factors and award assessment criteria that contribute to merit award-winning success. We do not believe that any one factor should be used alone as a predictor of future merit award-winning, by either medical schools or by individuals.

## Conclusions

Our original study uses national clinical award-winning as an outcome measure to add training and education data to the demographic description of successful doctors in Britain. Specifically, we determine and present the university medical schools which are most likely to generate award-winning pathologists. We also determine and present university medical schools most likely to generate award-winning non-pathologists. *This study is the first to calculate and present a ranking of university medical schools by the number of national award-winning pathologists.* Accordingly, we present comparative medical school data that can be used in the rational choice of medical schools for ambitious pathologist inclined, non-pathologist inclined and undecided medical school applicants.

We demonstrate that international medical graduates are making significant contributions to good pathology clinical practice in Britain, as judged by their concentration amongst the lower national merit award-winners. We provide evidence that indicates globalization and diversity of medical school origin are being reflected in the merit awards, indicating that Britain is a credible destination for ambitious medical trainees that seek national or international success.

## Data Availability

Data from this article is available upon reasonable request to the authors. Dr Sinclair Steele is the corresponding author and will make the data available https://www.sehd.scot.nhs.uk/publications/DC20200319SACDA.pdfhttps://www.gov.uk/government/publications/accea-annual-report-2020https://www.gmc-uk.org/registration-and-licensing/the-medical-registerhttps://olr.gdc-uk.org/SearchRegister.

## Abbreviations

IMG: International Medical Graduate
NCIA: National Clinical Impact Awards
CEA: Clinical Excellence Awards
DA: Distinction Awards
